# Interactions Among Morphology, Word Order, and Syntactic Directionality: Evidence from 55 Languages

**DOI:** 10.3390/e27111128

**Published:** 2025-10-31

**Authors:** Wenchao Li, Haitao Liu

**Affiliations:** 1 School of International Studies, Zhejiang University, Hangzhou 310058, China; 2 College of Foreign Languages and Literature, Fudan University, Shanghai 200437, China

**Keywords:** morphology, word order, syntactic directionality, typology, cross-linguistic variation

## Abstract

This study investigates interactions among morphology, word order, and syntactic directionality across 55 languages from 11 families. We quantify morphological richness (moving-average mean size of paradigm), word order flexibility (entropy), and syntactic directionality (dependency direction), linking linguistic structure to information-theoretic principles. Analyses show that morphological richness is only weakly related to word order entropy and does not provide a robust predictor after statistical correction. Rich morphology facilitates the predictability of syntactic functions. Languages with richer morphology consistently favor head-final structures, whereas minimally inflected languages lean toward head-initial patterns, indicating that syntactic directionality is more closely associated with morphological complexity than with surface word order. Overall, the findings indicate that languages maintain a balance between redundancy and flexibility in optimizing information transmission, providing quantitative evidence for efficiency-driven trade-offs in human language.

## 1. Introduction

Human language is a structured yet adaptive system governed by underlying regularities [1]. A central principle in linguistic typology is the complexity trade-off, which posits that when a language develops elaborate features in one domain—such as phonology, morphology, syntax, or semantics—it tends to simplify another, thereby maintaining an overall equilibrium in communicative complexity [2,3,4,5]. Finnish provides a canonical example of this trade-off. As a highly inflected language, it conveys dependency relationships through rich morphological markers, permitting flexible word order. For instance, the sentence “The boy reads a book” can be expressed in multiple orders: SVO (*Poika lukee kirjan*), SOV (*Poika kirjan lukee*), VSO (*Lukee poika kirjan*), VOS (*Lukee kirjan poika*), OSV (*Kirjan poika lukee*), and OVS (*Kirjan lukee poika*) (SVO: *Poika lukee kirjan* = poika.NOM boy, luke-a.3SG.PRS read, kirja-ACC book → ‘The boy reads the book.’ SOV: *Poika kirjan lukee* → ‘The boy reads the book.’ (object focus). VSO: *Lukee poika kirjan* → ‘It is the boy who reads the book.’ VOS: *Lukee kirjan poika* → ‘The boy reads the book.’ (subject focus). OSV: *Kirjan poika lukee* → ‘The book, the boy reads.’ OVS: *Kirjan lukee poika* → ‘It is the boy who reads the book.’). Thai, a Tai-Kadai language with minimal inflection, relies primarily on fixed word order and contextual cues to convey grammatical relations, following an SVO pattern: เด็กชาย อ่าน หนังสือ (*dèk-chaai àan năng-sĕu*; boy.NOM read.PRS book.ACC).

Recent advances in natural language processing and the availability of large-scale dependency treebanks have enabled quantitative investigation of cross-linguistic patterns [3]. Prior research has examined interactions among linguistic subsystems, including the interplay between word class shifts and morphological marking [6], the effect of morphology on lexicalization of speech act markers [7], the relationship between word order and case marking [8,9], the connection between syllable structure, phonemes, and morphology [10], the influence of verb-final order and case marking [11], as well as the impact of verb order, semantic closeness, and cognitive load [10,11,12]. These studies highlight that word order emerges from complex interactions among morphology, syntax, and information-processing constraints.

Within this framework, Hawkins’ efficiency-based theory of grammar offers a processing perspective on word order optimization. It posits that grammars tend to select and arrange linguistic forms so as to provide the earliest possible access to the developing syntactic–semantic representation during incremental parsing [13]. This Maximize Online Processing (MOP) principle links grammatical organization to processing efficiency, suggesting that constituent ordering reflects pressures to minimize integration cost and maximize early structure building. Building on this view, the present study investigates how morphological richness and dependency directionality interact under such processing efficiency constraints. Empirical evidence by Sinnemäki & Haakana [14] supports this perspective: in genitive noun phrases, richer morphological marking correlates with shorter dependency distances. However, their analysis was restricted to noun phrases and did not consider other syntactic constituents such as adjectives, determiners, relative clauses, adverbs, or full clauses. Consequently, the broader impact of morphological richness on syntactic directionality and word order flexibility remains underexplored.

The present study addresses this gap using 80 UD treebanks representing 11 language families. Specifically, we examine three variables: morphological richness (moving-average mean size of paradigm), word order flexibility (entropy), and syntactic directionality (dependency direction). The study is guided by two research questions:

**RQ1:** Does morphological richness correlate with word order flexibility? Rich morphology may provide cues that facilitate dependency parsing, potentially allowing greater variation in word order.

**RQ2:** Does morphological richness influence syntactic directionality? Languages with richer morphology may favor specific structural patterns, such as head-final or head-initial configurations, optimizing the transmission of information while reducing cognitive load.

By quantifying these relationships across a large typologically diverse sample, this study aims to provide quantitative evidence for efficiency-driven trade-offs in human language, shedding light on how morphological complexity interacts with syntactic structure to balance redundancy and flexibility.

## 2. Methodology

### 2.1. Data

The data used in this study comprised 55 languages, spanning 22 language branches across 11 language families. These families included Indo-European, Dravidian, Uralic, Altaic, Afro-Asiatic, Sino-Tibetan, Austronesian, Niger-Congo, and South Asian, as well as Basque and Japanese. Detailed information on the linguistic characteristics and the corresponding treebanks can be found in Appendix A and Appendix B. In Semitic languages such as Arabic and Hebrew, prepositions are often orthographically attached to nouns, forming a single word. However, for the purposes of dependency syntactic analysis, such constructions should be segmented into two distinct tokens, i.e., one representing the preposition and the other the noun. To ensure consistency and cross-linguistic comparability, all treebanks in this study employed the Universal Dependencies (UD) annotation framework [15]. UD systematically separates clitics and treats them as independent tokens, thereby minimizing errors arising from orthographic variation across languages. The UD framework provides part-of-speech tags, lemmas, and syntactic dependency trees for each word in the corpus. Each word is linked to a single syntactic head, and dependency relations are annotated using standardized labels such as *nsubj* (nominal subject), *obj* (direct object), and *amod* (adjectival modifier), among others. All metric computations and statistical analyses, including correlation tests between variables, were implemented using the Python programming language (version 3.11).

### 2.2. Metrics

This study evaluated three syntactic and morphological dimensions: morphological richness, word order flexibility, and syntactic directionality.

#### 2.2.1. Metric for Morphological Richness

A wide range of corpus-based metrics has been developed to capture morphological richness, including type–token ratio, word and lemma entropy, information-theoretic measures, and paradigm-based counts [16]. While each metric offers a distinct perspective, many are sensitive to corpus size, structural domains (e.g., verbal vs. nominal), or text genre, making cross-linguistic comparison difficult. To address this, the present study adopts a paradigm-oriented measure inspired by Xanthos and Gillis [17]: the mean size of paradigm (MSP), which captures the average number of inflected forms per lemma. To enhance robustness across corpora of varying lengths and syntactic densities, we further refined this metric using a moving-average framework, resulting in the moving-average mean size of paradigm (MAMSP). This approach mitigates the impact of local fluctuations by computing paradigm size within fixed-size token windows. Formally, MAMSP is defined as follows:

(1) MAMSP $=\frac{\sum_{i=1}^{N-W+1}\frac{F_{i}}{Li}}{N-W+1}$
  where *N* refers to the number of text tokens in the data. *W* represents the window size, where *W < N*. *Fi* denotes the number of distinct inflected word forms in each window. *Li* represents the number of distinct word lemmas in each window. To illustrate, consider the following Japanese sentence:
(2) 朝早く駅に着いて彼女［が omitted］来るのを
  Morning early ADV station-DAT arrive-GER she-NOM [omitted] come-NMLZ-ACC
  待ったけど、電車［が omitted］遅れて会えなかった.
  wait-PST CONJ train-NOM [omitted] late-GER meet-POT-NEG-PST
  “I arrived early at the station and waited for her to come, but the train was late, so I could not meet her.”

This sentence contains 11 tokens (N = 11). Suppose the window size is W = 5. Then, we can slide the window to obtain N – W + 1 = 7 windows: 朝, 早く, 駅に, 着いて, 彼女; 早く, 駅に, 着いて, 彼女, 来るのを; 駅に, 着いて, 彼女, 来るのを, 待った; 着いて, 彼女, 来るのを, 待った, けど; 彼女, 来るのを, 待った, けど, 電車; 来るのを, 待った, けど, 電車, 遅れて; 待った, けど, 電車, 遅れて, 会えなかった. For each window, compute the ratio Fi/Li, i.e., the number of distinct inflected word forms divided by the number of distinct lemmas. The final MAMSP is the average of these seven ratios:

(3) MAMSP = $\frac{\sum_{i=1}^{N-W+1}\frac{F_{i}}{Li}}{N-W+1}$= $\frac{\frac{1}{2}+\frac{1}{3}+\frac{1}{3}+\frac{1}{2}+\frac{2}{3}+\frac{1}{2}+\frac{1}{1}}{11-5+1}$ ≈ 0.548

This moving-average approach smooths local fluctuations and provides a stable estimate of morphological richness across different corpus lengths.

#### 2.2.2. Metric for Word Order Flexibility

Word order is influenced by a multitude of linguistic factors, including adpositions, genitives, noun modifiers, demonstratives, numerals, adjectives, relative clauses, and adverbs, among others [18]. For the purpose of this study, our attention is centered on the sequence of the constituents: subject (S), object (O), and verb (V). This choice is justified by two main considerations: (a) S, O, and V are central syntactic elements that appear in the vast majority of sentences across different dialects, making them ideal for typological comparison, and (b) their identification is relatively straightforward across varied language datasets [19]. We considered the six canonical word orders, i.e., SVO, OVS, VSO, VOS, SOV, OSV, as well as partial orders such as SV, VO, and SO, which arise due to intransitive clauses, ellipsis, copula-drop, or nominal predications. Our Python-based tool extracts these linearizations directly from dependency structures, allowing the analysis to accommodate varied syntactic patterns and reflect actual language use. While some languages demonstrate strict word order constraints in subordinate clauses or coordinated structures [20], this study focuses exclusively on main clauses.

In measuring word order flexibility, several metrics have been proposed, such as maximum–minimum distance, Euclidean distance, cosine similarity, and entropy [19]. Among these, Kubon et al. found a convergence in their ability to capture variability, suggesting any of these can serve as reliable indicators. Our study adopts entropy as the principal metric due to its interpretability and prevalence in recent linguistic research on syntactic variation [6,21]. Entropy (ENTR), the metric selected for this study, captures the randomness or unpredictability of word order use [22]. It is defined as follows:

(4) ENTR = $-\sum_{i=1}^{6}s_{i}\;ln\;s_{i}$

$s_{i}$ denotes the relative frequency of the *i*-th word order type, including both canonical (SVO, OVS, etc.) and partial orders (SV, VO, SO, etc.), with $s_{i}$ ≥ 0 and $\sum_{i}s_{i}$ = 1. The index iii thus ranges over all attested word order variants. Entropy reaches its maximum when all word orders are equally probable, yielding ln 6 ≈ 1.794, and decreases as distributions become more skewed toward fewer dominant orders. By employing entropy in this way, our metric captures the degree of flexibility in the linear arrangement of syntactic elements, accommodating both complete and partial clause structures.

#### 2.2.3. Metric for Syntactic Directionality

Syntactic directionality reflects the linear order between syntactic heads and their dependents. Cross-linguistically, some languages tend to favor head-initial structures, where the head precedes its dependent (as in English), while others prefer head-final structures, such as Japanese. Many languages display mixed or flexible ordering patterns [23,24]. In this study, we operationalized syntactic directionality in terms of dependency directionality. To capture this structural property, we adopted the dependency direction (DDir) score [20,23], a quantitative metric that summarizes the global syntactic ordering tendency of a language. It was computed over all dependency relations in a syntactically annotated corpus (treebank). For each dependency arc, we determined whether the head precedes or follows its dependent and then calculated the ratio between head-initial and head-final dependencies. Formally, the DDir score is defined as follows:

(5)
$DDir=\frac{NHI-NHF}{Ntotal}$

This definition normalizes the difference between head-initial (NHI) and head-final (NHF) dependencies by the total number of dependencies (Ntotal), yielding values between −1 and +1. A score close to +1 indicates a strongly head-initial language, a score near −1 suggests a predominantly head-final language, and values around 0 imply mixed directionality.

It is important to note that the DDir measure is based on head-dependent direction in the dependency tree, not on surface word order. For example, in English, both the active sentence *‘I ate an apple’* and its passive counterpart *‘An apple is eaten by me’* have the same head-dependent directions: the verb is the head, and both subject and object (or agent in the passive) are dependents. Therefore, passive constructions do not affect the dependency direction statistics in our UD-based analysis.

Furthermore, to assess the robustness of the three metrics across different data sources (treebanks), we conducted a pilot test by calculating MAMSP, ENTR, and DDir using multiple treebanks for three languages: German, Tamil, and Turkish. We compared German-HDT vs. German-GSD, Tamil-TTB vs. Tamil-MWTT, Turkish-Kenet vs. Turkish-Boun. Results revealed moderate variation. For German, DD_ir_ (0.6356 vs. 0.6576) and MAMSP (0.982 vs. 0.9622) are relatively close, indicating consistency in morphological annotation and dependency structure. ENTR shows greater divergence (1.157 vs. 1.3439), suggesting that syntactic variability is more corpus-sensitive. For Tamil, MAMSP remains stable (0.9995 vs. 0.9763), but DD_ir_ (0.6463 vs. 0.7105) and especially ENTR (0.1391 vs. 0.6663) vary substantially, implying differences in how word order patterns are captured. For Turkish, the two treebanks produce relatively consistent values across all three metrics, with only minor differences. These findings indicate that morphological richness is generally stable across treebanks, while word order flexibility is more sensitive to genre, domain, and annotation guidelines. Dependency relation directionality (DD_ir_) shows moderate variation. In light of this, we adopted a triangulation strategy to improve the reliability of our linguistic measurements. For languages with multiple treebanks, we computed each metric separately for each source. If results were consistent, we treated them as robust. If they diverged, we calculated the mean across treebanks to reduce bias.

### 2.3. Units of Analysis

The present study investigates cross-linguistic relationships among morphological richness, word order flexibility, and syntactic directionality. The intended level of inference is typological (i.e., across languages), whereas the empirical level of observation is corpus-based. To balance representativeness and statistical independence, each UD tree bank is treated as a single analytical unit. As summarized in Appendix B, some languages (e.g., German, Japanese, Turkish) are represented by multiple treebanks differing in domain and genre. Treating each treebank as one observation prevents languages with multiple treebanks from being overrepresented and avoids within-language autocorrelation. Accordingly, all statistical analyses (including ANOVA and Pearson correlation tests) were conducted using per-treebank mean scores as independent data points. Each tree bank contributed one averaged value per metric (MAMSP, ENTR, and DDir).

## 3. Results

### 3.1. Morphological Richness

**Figure 1:**
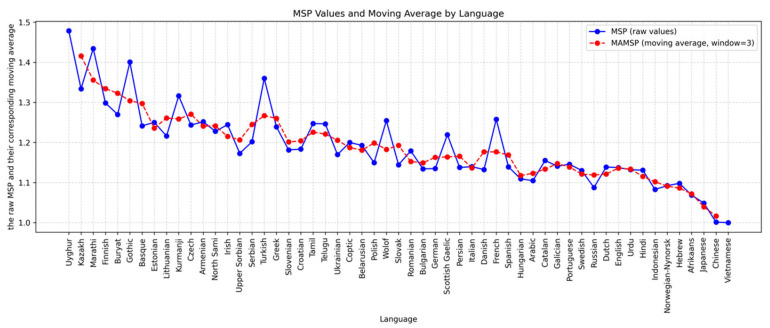
Raw MSP and moving averages across languages.

Appendix C summarizes the MAMSP values for each language, where higher scores indicate greater morphological richness. Figure 1 illustrates both the raw MSP values (blue) and their moving averages (red).

A one-way ANOVA tested differences in MAMSP across 22 language branches, using per-treebank mean MAMSP scores as independent data points. The analysis revealed a significant effect of language branch on morphological richness (F (21, 33) = 2.470, *p* = 0.0097, η^2^ = 0.611, ω^2^ = 0.360). Based on MAMSP (the smoothed measure), the ten morphologically richest languages are predominantly agglutinative, including Uyghur and Kazakh (Turkic branch, Altaic family), Marathi (Indo-Aryan, Indo-European), Finnish and Estonian (Finno-Ugric, Uralic), Buryat (Mongolic, Altaic), Kurmanji (Iranian, Indo-European), Tamil and Telugu (Dravidian), and Wolof (Atlantic, Niger-Congo). These results confirm that agglutinative systems, which express grammatical meaning through concatenated, segmentable morphemes, tend to yield a larger number of distinct surface forms per lemma—hence higher MAMSP values. This correlation aligns with typological findings in the literature [17,25,26,27]. French and Gothic, which rank relatively high in *raw* MSP but not in MAMSP, illustrate that inflectional irregularities and portmanteau morphemes (e.g., *je vais*, *tu vas*) can temporarily inflate raw morphological counts. The MAMSP-based results, therefore, provide a more stable and typologically coherent measure of morphological richness across language families.

Within-family contrasts further support these trends. In the Uralic family, the Finnic languages (Finnish, Estonian) show significantly higher MAMSP scores than Finno-Ugric members (Hungarian, North Sámi): *t* = −12.83, *p* = 1.76 × 10^−36^. In the Indo-European family, Polish is morphologically richer than Ukrainian (*t* = −3.76, *p* = 0.00056), and Marathi, the only agglutinative Indo-Aryan language, exceeds Urdu and Hindi in richness. Within the Altaic family, Turkic languages exhibit significantly higher MAMSP values than Mongolic languages (*t* = 3.77, *p* = 0.00017). At the lower end of the scale, Chinese and Vietnamese show minimal MAMSP values, consistent with their isolating morphological type. Languages from the Celtic, Basque, Uralic, Baltic, and Slavic branches display moderate morphological richness, while most Romance and Germanic languages are comparatively low. Interestingly, Japanese, despite its agglutinative character, ranks lower than expected. This likely results from its limited nominal inflection—grammatical relations are encoded primarily by independent case particles (が, を, に) rather than by affixal morphology. Because the MAMSP metric quantifies affix-based morphological productivity, Japanese appears less morphologically rich under this measure. This effect is reinforced by the UD annotation scheme, which treats Japanese particles as separate tokens rather than bound morphemes.

### 3.2. Syntactic Directionality

**Figure 2:**
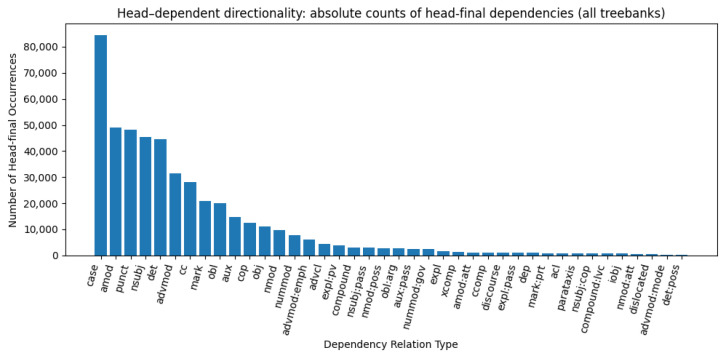
Head-dependent directionality: absolute counts of head-final dependencies across relation types (≈856 k dependencies in total; 38.5% head-final).

Languages with greater morphological richness show a tendency toward head-final dependency structures (Pearson r = −0.369, 95% CI [−0.611, −0.085], *p* = 0.0055). Languages with high MAMSP values, such as Uyghur, Marathi, Turkish, Kazakh, Kurmanji, Buryat, Tamil, and Telugu, exhibit a strong preference for head-final dependencies. Across all treebanks, we analyzed a total of ≈856,000 dependency relations (excluding ROOT). Among these, ≈330,000 were head-final (38.5%) and ≈526,000 were head-initial (61.5%). Figure 2 summarizes absolute counts of head-final occurrences by dependency relation type, aggregated across all treebanks. For interpretability, Appendix D reports, for each relation, both (i) the within-relation head-final percentage and (ii) the share of the full corpus. For example, for nmod, there are 9688 head-final instances, which correspond to 13.84% of all nmod relations (9688/69,981) and about 1.1% of all dependencies in the dataset (9688/856,000). Consistent head-final behavior is observed for *case*, *det*, *cc*, *mark*, *amod*, *advmod*, *nsubj*, and *aux*, particularly within morphologically rich (high-MAMSP) languages. By contrast, relations such as *obj*, *obl*, *acl:relcl*, *xcomp*, and *nmod* show lower head-final proportions in the aggregate. These distributional patterns align with typological observations that languages with more robust inflectional/agglutinative morphology tend to favor head-final sequencing in core phrasal domains [9,25], while also reflecting cross-branch mixing in relations sensitive to clause structure (*obj*, *obl*, *xcomp*). Overall, head-final dependencies constitute a numerical minority in the aggregated corpus, yet they are typologically concentrated in morphologically rich languages with predominantly head-final syntactic structures.

### 3.3. Word Order Flexibility

Appendix E reports the ENTR values for all languages, where higher scores reflect greater freedom in constituent ordering. A one-way ANOVA tested branch-level differences in ENTR, using per-treebank mean ENTR scores as independent observations (F (21, 33) = 2.13, *p* = 0.025). The analysis yielded a moderate-to-large effect size (η^2^ = 0.576, ω^2^ = 0.302), indicating that language branch accounts for a substantial share of the observed variation in word order flexibility. The top five most flexible languages are as follows: agglutinative: Wolof (Atlantic), Kurmanji (Iranian); inflectional: Lithuanian (Baltic), Slovak, Czech (Slavic). These are followed by Finnic, Slavic, Germanic, and Basque languages. In contrast, Indo-Aryan, Celtic, Sinitic, and Vietic languages exhibit more rigid word order. Appendix F further provides the distribution proportions of six word-order types across 55 languages. Among these, Lithuanian stands out with a high degree of inflectional variation and the most extensive case system among Indo-European languages. This rich inflectional variation and case system contribute to Lithuanian having the most relaxed word order. Kurmanji, an agglutinative language, ranks second in word order freedom. Czech, Slovak, Slovenian, German, Finnish, and Hungarian demonstrate the most balanced proportions of S, V, and O combinations. The Slavic language family showcases all possible word orders, with preferences in the order of SVO > OVS > VOS > SOV > VSO > OSV. Among the eleven Slavic languages, Czech, Slovak, and Slovenian exhibit the most balanced distribution of word order types. This explains why these three languages have higher ENTR values compared to other Slavic languages. Within the Uralic sample examined here (Finnish, Estonian, Hungarian, and North Sámi), all six canonical word orders are attested in the UD treebanks. Among them, SVO is the most frequent pattern overall, though its proportion varies substantially across languages. Estonian shows a strong SVO preference (≈85%), Hungarian favors SVO (≈59%) but also displays considerable SOV and OVS variation, while Finnish exhibits the most flexible ordering, with SVO ≈ 41% and a notably high proportion of OSV and OVS (≈38%). The Altaic language family exhibits all word orders but has a preference for SOV. The Turkic language family (Turkish, Kazakh, Uyghur) shows a word order preference of SOV (78%) > OVS (15%) > SVO (3%) > OSV (4%) > VSO (1%) > VOS (0%). Within the Altaic language family, Mongolic Buryat shows a higher proportion of SVO (27.45%) and a lower proportion of OVS. This is due to the influence of Slavic languages (dominated by SVO) on the Buryat language, despite it being part of the Mongolic language family and administratively belonging to one of the autonomous republics of the Russian Federation. This explains why the word order preference of Buryat is not related to the Altaic type but clusters with Slavic languages.

The Afro-Asiatic Semitic family exhibits distinct word order preferences. Arabic and Maltese strongly favor verb-initial (VSO) patterns (about 70%), reflecting a consistent syntactic profile. Modern Hebrew shows more variable distributions: fully realized SVO and VSO clauses are relatively infrequent, while a large share of clauses contain only two constituents (SV, VS, VO, or OV). This structural variability contributes to Hebrew’s higher word order entropy compared to Arabic and Maltese. Table 1 and Table 2 summarize the distribution of major word orders in these three languages, based on UD treebank data.

Irish is a verb-initial language, with the VO pattern accounting for 98%. The Afro-Asiatic Egyptian language family’s Coptic language, which is agglutinative, exhibits more word order variation than the Semitic languages in the same family. Marathi, Urdu, and Hindi show slightly more flexibility in word order compared to Arabic, tending towards SOV > SVO > OSV. In the previous analysis of morphological richness, Marathi demonstrated the highest diversity among Indo-Aryan languages, and in this word order test, it exhibits the highest flexibility as well. This observation is consistent with the tendency that languages with richer morphology often display greater flexibility in constituent order. Additionally, the Dravidian languages Telugu and Tamil, belonging to the Dravidian language family, exhibit moderate rigidity in word order and a preference for verb-final structures (cf. SOV > OSV > OVS).

Looking at the Indo-European language family, most of the 34 languages examined in this study show a preference for SVO. However, German, Dutch, Gothic, and Greek display verb-initial word order (VOS, VSO) in interrogative and negative sentences. Within the Romance language family, Galician primarily employs SVO but combines it with a certain proportion of VSO and SOV. Basque is an agglutinative language with a balanced distribution of word orders, preferring SVO (58.84%) > SOV (30.39%) > OSV (4.76%) > OVS (3.97%) > VOS (1.70%) > VSO (0.34%). Armenian, while morphologically similar to Turkish, with suffixes used to indicate grammatical information, differs from Turkish in word order preference. Armenian primarily favors SVO and exhibits all possible word orders. Finnish, due to its rich derivation and inflectional morphology, enjoys relatively free word order. The Germanic language family prefers SVO > VSO > OVS. English, within the Germanic language family, is the least flexible in word order, strongly favoring the SVO pattern.

### 3.4. Cross-Linguistic Correlations

To synthesize the patterns observed in Section 3.1, Section 3.2 and Section 3.3, we performed Pearson correlation analysis among the three metrics: MAMSP (morphological richness), DDir (dependency direction), and ENTR (word order flexibility). Each UD treebank contributed one averaged value per metric, which served as an independent observation in the cross-linguistic correlation tests. The following correlations were observed: MAMSP vs. DDir: r = −0.370, 95% CI [−0.62, −0.09], *p* = 0.005. MAMSP vs. ENTR: r = 0.267, 95% CI [0.00, 0.50], *p* = 0.049. DDir vs. ENTR: r = 0.013, 95% CI [−0.27, 0.30], *p* = 0.924. After correction for multiple comparisons, the MAMSP–DDir correlation remained significant under both Bonferroni and FDR procedures, while the MAMSP–ENTR trend did not. DDir–ENTR showed no relationship under any threshold.

These results show that morphological richness is negatively correlated with head-initial directionality—languages with richer morphology tend to favor head-final structures—while its positive association with word order flexibility is weak and not statistically robust. No meaningful correlation is found between syntactic directionality and word order flexibility.

**Figure 3:**
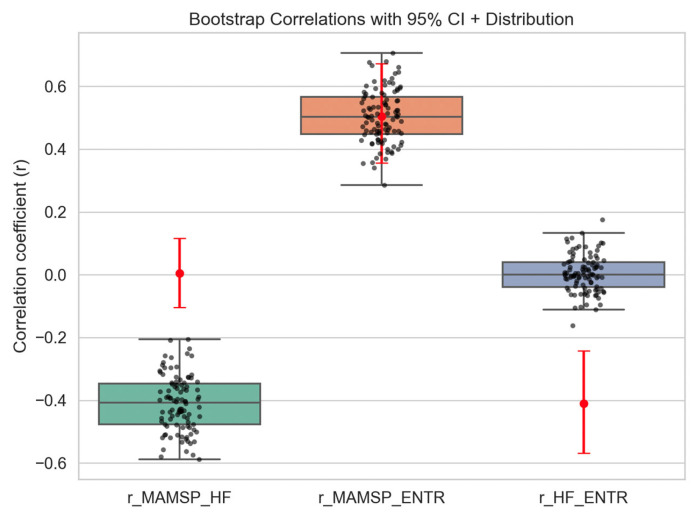
Bootstrap distribution of correlation coefficients for r_MAMSP_HF, r_MAMSP_ENTR, and r_HF_ENTR.

To account for potential biases due to genealogical non-independence among languages, we performed a one-per-family bootstrap (100 iterations) for these three pairwise correlations. Figure 3 presents the bootstrap distributions: r_MAMSP_HF: median ~ −0.5, 95% CI mostly negative, confirming a robust negative correlation; r_MAMSP_ENTR: median ~0.5, 95% CI overlaps zero, indicating a non-significant positive trend. r_HF_ENTR: median ~0, 95% CI spans zero, confirming no significant relationship between head-final dependency preference and word order flexibility. These results reinforce that the negative correlation between morphological richness and head-final preference is robust, while positive trends between morphology and word order flexibility are inconclusive, and syntactic directionality and word order flexibility are uncorrelated.

## 4. Discussion: Interactions Among Morphology and Syntactic Directionality

The central finding of this study is that morphological richness reliably predicts dependency directionality, whereas its association with word order flexibility is weaker. Languages with richer inflectional systems consistently favor head-final dependency structures, and this effect remained significant even under conservative Bonferroni correction, reinforcing the view that morphological richness plays a stable role in shaping dependency directionality across languages. The effect size was moderate (r ≈ −0.37), indicating a substantial but not deterministic influence of morphology on syntactic organization. This result is consistent with Dryer [28] in that our measure of syntactic directionality (DDir) captures the same type of head-directional tendencies he reported for OV and VO languages, for example, the association of OV with RelN and VO with NRel structures. Our analysis builds on Dryer’s typological framework by extending it into a quantitative, corpus-based approach that integrates morphological richness (MAMSP) with syntactic directionality.

The observed correlation between morphological richness and head-finality may reflect a structural alignment between morphological and syntactic organization. In most languages, morphological complexity is largely realized through suffixation, and suffixes are often analyzed as syntactic heads. From this perspective, rich morphology, being predominantly suffixing, can be viewed not merely as an independent correlate but as a morphological manifestation of head-final organization. In other words, the same underlying head-final principle that governs syntactic ordering may also shape morphological structure. This interpretation suggests that the correlation is not accidental but reflects a cross-level structural consistency between morphology and syntax. Future research could empirically test this account by quantifying the cross-linguistic balance between suffixing and prefixing patterns.

**Figure 4:**
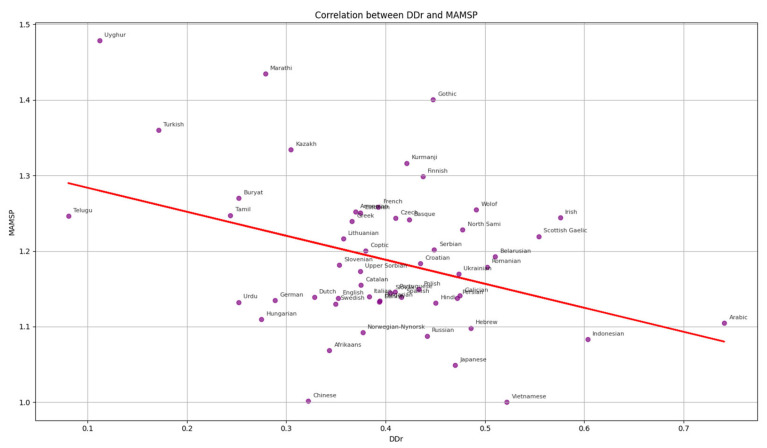
Scatter plots of morphological richness and syntactic directionality.

**Figure 5:**
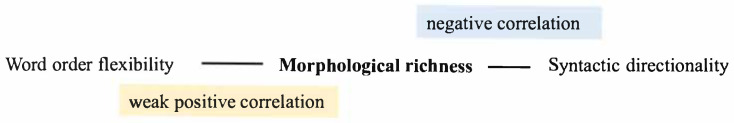
Network of morphological and syntactic subsystems.

The correlation between morphological richness and word order flexibility was weaker and did not remain significant after correction for multiple comparisons. This suggests that morphology may sometimes enable freer constituent order, but such effects are less stable and context-dependent. No evidence was found for a correlation between word order flexibility and dependency directionality. This pattern is broadly consistent with typological observations under the OV/VO contrast, which indicate that while certain structural correlations are robust, others may be region- or language-specific rather than universal. Figure 4 visualizes raw bivariate trends between morphological richness and syntactic directionality, while Figure 5 presents the network of dependencies, showing that morphology exerts a primary influence on syntax, whereas word order flexibility plays a more peripheral role.

While our results largely support Dryer’s observations, it is worth noting that Benítez-Burraco et al. [29] report a contrasting finding, suggesting that there may be no systematic trade-off between morphological and syntactic complexity across languages. The differences between their results and ours may be partly attributable to variations in data sources, methodology, and metrics. Our study relies on annotated treebanks from 55 languages across 11 language families, using per-treebank measurements of dependency direction (DDir), word order flexibility (ENTR), and morphological richness (MAMSP). Benítez-Burraco et al. use the cross-linguistic typological database (WALS), which aggregates language-level features. Furthermore, our metrics quantify syntactic linearity and dependency structures, capturing more subtle interactions between morphology and syntax in naturalistic language use. Their indicators focus on broader typological categories and feature counts, which may not distinguish head-dependent directionality or word order variability. Finally, our analysis applies statistical correlations and ANOVA on per-treebank measures, which may be sensitive to micro-level co-variation patterns. Taken together, the discrepancy likely stems from differences in data granularity and methodological scope rather than genuine theoretical conflict. Within our corpus-based framework, we observe that languages with richer morphology favor head-final dependencies and show a mild, non-significant trend toward greater word order flexibility, which may indicate a subtle interaction between morphological richness and syntactic structure that emerges in annotated corpus data.

Recalling Section 2.2.3, to assess the robustness of the three metrics across different treebanks, we compared German-HDT vs. German-GSD, Tamil-TTB vs. Tamil-MWTT, and Turkish-Kenet vs. Turkish-Boun. The results revealed that word order flexibility is more sensitive to factors such as genre, domain, and annotation scheme. Still, one would argue that for languages with “free word order,” such flexibility is not absolute; word order often carries pragmatic meaning related to information packaging, such as focus, topic, or stylistic considerations. For example, in Russian, which exhibits relatively free word order, the most important or emphasized element is typically placed first or last. A comparable example in English is the sentence “YOU, I do not understand,” which deviates from typical word order to emphasize “YOU.” These patterns may be influenced by genre or content/topic (e.g., poetry vs. news). The differences in genre and content composition within the corpora used in this study may influence the word order metrics. In languages characterized by ‘free word order,’ variation in constituent order often reflects information–structural adjustments, such as the arrangement of focus and topic. Therefore, preferences for word order use vary across genres, such as news reporting, spontaneous spoken dialogue, and poetry, impacting the statistical expression of dependency directionality and word order flexibility. Our analyses were based on the overall data from each treebank and did not distinguish between genres or pragmatic functions. This limitation may have partially obscured the influence of pragmatic factors on the word order metrics. Addressing this issue will require finer-grained analysis. Future studies could examine genre- or pragmatics-specific word order preferences to refine these metrics. While the present study treats each UD treebank as an independent corpus (per-treebank design), future research focusing on intra-language variation, such as stylistic, genre, or register effects, could adopt a per-document framework to capture subcorpus-level dynamics. Such an extension would provide a complementary, fine-grained perspective to the typological approach taken here.

## 5. Summary and Future Work

This study investigated the interactions among morphological richness, word order flexibility, and syntactic directionality across 55 languages from 11 major families. Using three quantitative metrics, i.e., MAMSP (morphological richness), ENTR (word order flexibility), and DDir (dependency direction), we examined how morphology relates to syntactic structure. Our results indicate that morphological richness strongly predicts dependency directionality, whereas its link to word order flexibility remains weaker and context-dependent. Substantial intrafamilial variation was observed; for instance, Finnic languages (e.g., Finnish, Estonian) exhibit more complex morphology than Finno-Ugric languages (e.g., Hungarian), and Turkic languages generally show richer inflection than Mongolic counterparts.

These findings align with Dryer’s typological generalizations. Consistent with his observation that OV languages often exhibit head-final dependencies, we found a moderate negative correlation between MAMSP and DD_ir_ (r ≈ −0.37, *p* = 0.005). Similarly, the weak trend linking morphological richness to word order flexibility resonates with Dryer’s point that certain structural tendencies, such as RelN/NRel patterns, manifest more strongly in some language types but are not universal. Our corpus-based, per-treebank measures complement Dryer’s genus- and language-level analysis by quantifying effect sizes, capturing confidence intervals, and revealing fine-grained variation in dependency direction and word order.

Several methodological and empirical considerations need attention. First, word order flexibility is sensitive to genre, domain, and annotation scheme, as shown by comparisons across multiple treebanks for German, Tamil, and Turkish. Second, current NLP pipelines differ in tokenization strategies (e.g., Stanza splits the French aux into à + les), which may affect cross-linguistic comparability. Third, the language sample remains typologically imbalanced, with some major families underrepresented, such as Austronesian, Sino-Tibetan, Indo-Aryan, and Niger-Congo, and features such as syllable structure, mean word length, and pragmatic constraints were not incorporated. Future work should address these limitations by expanding typological coverage, integrating genre- and pragmatics-specific analyses, and standardizing tokenization and syntactic annotation frameworks across corpora. Through such efforts, we can deepen our understanding of the interaction between grammar and cognition, and build more robust models of language as a complex adaptive system. Beyond linguistic theory, the findings also have implications: the observed trade-offs between morphology and syntax can inform typological modeling, enhance natural language processing systems by guiding cross-linguistic parser design.

## Figures and Tables

**Table 1:** Word order distribution in Semitic languages (full clauses).

Language/Data Type	SVO (%)	SOV (%)	VSO (%)	VOS (%)	OVS (%)	OSV (%)
Arabic (PUD + NYUAD)	18.1	0.0	72.8	NA	3.9	0.2
Maltese (MUDT)	24.1	0.0	69.9	NA	4.1	1.9
Hebrew-HTB	16.6	0.0	3.0	0.6	1.1	0.0
Hebrew-IAHLTwiki	19.6	0.0	10.0	0.0	0.8	0.0

**Table 2:** Word order distribution in Hebrew (partial clauses).

Language/Data Type	SV (%)	VS (%)	VO (%)
Hebrew-HTB	44.5	25.4	8.3
Hebrew-IAHLTwiki	40.0	24.8	4.4

## Data Availability

The raw data used in this study are openly available from the Universal Dependencies (UD) treebanks at https://universaldependencies.org. All metrics and values reported in this study were computed independently using custom scripts developed by the authors.

## Appendix A. Language Information

**Language Family**	**Branch**	**Languages**	**Morphology**	**Language Family**	**Branch**	**Languages**	**Morphology**
Indo-European	Germanic	Afrikaans	Fusional	Indo-European	Slavic	Croatian	Fusional
Indo-European	Germanic	Dutch	Fusional	Indo-European	Slavic	Slovenian	Fusional
Indo-European	Germanic	Norwegian-Nynorsk	Fusional	Indo-European	Slavic	Serbian	Fusional
Indo-European	Germanic	English	Fusional	Indo-European	Slavic	Upper Sorbian	Fusional
Indo-European	Germanic	Danish	Fusional	Indo-European	Slavic	Czech	Fusional
Indo-European	Germanic	German	Fusional	Indo-European	Greek	Greek	Fusional
Indo-European	Germanic	Swedish	Fusional	Indo-European	Baltic	Lithuanian	Fusional
Indo-European	Germanic	Gothic	Fusional	Dravidian	Dravidian	Telugu	Agglutinative
Indo-European	Indo-Aryan	Hindi	Fusional	Dravidian	Dravidian	Tamil	Agglutinative
Indo-European	Indo-Aryan	Urdu	Fusional	Uralic	Finno-Ugric	Hungarian	Agglutinative
Indo-European	Indo-Aryan	Marathi	Agglutinative	Uralic	Finno-Ugric	North Sami	Agglutinative
Indo-European	Romance	Portuguese	Fusional	Uralic	Finnic	Estonian	Agglutinative
Indo-European	Romance	Galician	Fusional	Uralic	Finnic	Finnish	Agglutinative
Indo-European	Romance	Catalan	Fusional	Altaic	Mongolic	Buryat	Agglutinative
Indo-European	Romance	Spanish	Fusional	Altaic	Turkic	Kazakh	Agglutinative
Indo-European	Romance	French	Fusional	Altaic	Turkic	Uyghur	Agglutinative
Indo-European	Romance	Romanian	Fusional	Altaic	Turkic	Turkish	Agglutinative
Indo-European	Romance	Italian	Fusional	Afroasiatic	Semitic	Hebrew	Fusional
Indo-European	Iranian	Kurmanji	Agglutinative	Afroasiatic	Semitic	Arabic	Fusional
Indo-European	Iranian	Persian	Fusional	Afroasiatic	Egyptian	Coptic	Agglutinative
Indo-European	Celtic	Scottish Gaelic	Fusional	Sino-Tibetan	Sinitic	Chinese	Isolating
Indo-European	Celtic	Irish	Fusional	Japanese	Japanese	Japanese	Agglutinative
Indo-European	Slavic	Bulgarian	Fusional	Austronesian	Malayo-Polynesian	Indonesian	Agglutinative
Indo-European	Slavic	Russian	Fusional	Armenian	Armenian	Armenian	Agglutinative
Indo-European	Slavic	Slovak	Fusional	Basque	Basque	Basque	Agglutinative
Indo-European	Slavic	Polish	Fusional	Niger-Congo	Atlantic	Wolof	Agglutinative
Indo-European	Slavic	Belarusian	Fusional	Austroasiatic	Vietic	Vietnamese	Isolating
Indo-European	Slavic	Ukrainian	Fusional				

## Appendix B. Treebank Information

**Language Branch**	**Treebanks**	**Text Types**	**Words**	**Sentences**
Armenian	Armenian-ArmTDP	Blog, fiction, grammar-examples, nonfiction, news, legal	52,585	2500
Armenian	Armenian-BSUT	Blog, fiction, government, web, wiki, nonfiction, news, legal	41,805	2300
Basque	Basque-BDT	News	121,443	8993
Germanic	Afrikaans-AfriBooms	Legal nonfiction	49,260	1934
Germanic	German-HDT	News, nonfiction, web	3,455,580	189,928
Germanic	German-GSD	Review, wiki, news	292,769	15,590
Germanic	Danish-DDT	Fiction, nonfiction, news, spoken	100,733	5512
Germanic	Dutch-Alpino	News	208,748	13,603
Germanic	Dutch-LassySmall	Wiki	98,241	7341
Germanic	English-GUM	Academic, blog, email, fiction, government, grammar-examples, legal, medical, news, nonfiction, poetry, reviews, social, spoken, web, wiki	187,515	10,761
Germanic	Swedish-Talbanken	News, nonfiction	96,859	6026
Germanic	Swedish_LinES	Spoken, fiction, nonfiction	90,960	5243
Germanic	Swedish-PUD	News, wiki	19,085	1000
Germanic	Gothic	Bible	55,336	5401
Germanic	Norwegian-Nynorsk	Blog, news, nonfiction	301,353	17,575
Slavic	Slovenian-SSJ	Fiction, news, nonfiction	267,097	16,623
Slavic	Spoken Slovenian	Spoken	29,488	3188
Slavic	Ukrainian-IU	Blog, email, fiction, grammar-examples, legal, news, reviews, social, web, wiki	122,983	7092
Slavic	Serbian-SET	News	97,673	4384
Slavic	Belarusian-HSE	Fiction, legal, news, notification, web, social, wiki	305,417	25,231
Slavic	Bulgarian-BTB	Fiction, legal, news	156,149	11,138
Slavic	Croatian-SET	News, web, wiki	199,409	9010
Slavic	Czech-CAC	Fiction, legal, medical, news, nonfiction, wiki, reviews	495,497	24,709
Slavic	Czech-PDT	News, nonfiction, reviews	1,530,008	87,907
Slavic	Polish-LFG	Fiction, news, social, spoken, nonfiction	130,967	17,246
Slavic	Russian-Taiga	Fiction, news, wiki, blog, email, nonfiction, poetry, social	197,001	17,872
Slavic	Slovak-SNK	Fiction, news, nonfiction	106,184	10,604
Slavic	Upper Sorbian-UFAL	Wiki, nonfiction	11,196	646
Japanese	Japanese-BCCWJ	Fiction, news, blog, conference, nonfiction	1,253,903	57,109
Dravidian	Tamil-TTB	News	9581	600
Dravidian	Tamil-MWTT	News	2584	534
Dravidian	Telugu_MTG	Grammar-examples	6465	1328
Altaic	Buryat-BDT	Grammar examples, news, fiction	10,185	927
Altaic	Kazakh-KTB	News, fiction, wiki	10,536	1078
Altaic	Turkish-Kenet	News, nonfiction	183,555	16,396
Altaic	Turkish-Boun	News, nonfiction	125,212	9761
Altaic	Uyghur_UDT	Fiction	40,236	3456
Romance	Catalan-AnCora	News	553,042	16,678
Greek	Greek-GUD	Grammar examples	25,493	1807
Greek	Greek-GDT	Wiki, news, spoken	63,441	2521
Romance	French-Rhapsodie	Spoken	44,242	3209
Romance	French-Paris Stories	Spoken	42,795	2776
Romance	French-GSD	Blog, news, review, wiki	400,489	16,342
Romance	Spanish-PUD	News, wiki	23,287	1000
Romance	Spanish-AnCora	News	567,894	17,662
Romance	Spanish-GSD	Blog, news, review, wiki	431,584	16,013
Romance	Galician-TreeGal	News	25,548	1000
Romance	Italian-VIT	News, nonfiction	280,154	10,087
Romance	Portuguese-Bosque	News	227,827	9357
Romance	Portuguese-PUD	News, wiki	23,407	1000
Romance	Romanian-RRT	Academic, legal, fiction, medical, nonfiction, news, wiki,	218,522	9524
Indo-Aryan	Hindi-HDTB	News	351,704	16,649
Indo-Aryan	Hindi-PUD	News, wiki	23,829	1000
Indo-Aryan	Marathi-UFAL	Wiki, fiction	3847	466
Indo-Aryan	Urdu-UDTB	News	138,077	5130
Baltic	Lithuanian-ALKSNIS	News, fiction, nonfiction, legal	70,051	3642
Baltic	Lithuanian-HSE	News, nonfiction	5356	263
Celtic	Irish-IDT	News, web, fiction, government, legal	115,990	4910
Celtic	Irish-twitter	Social	47,790	2596
Celtic	Scottish Gaelic	Fiction, news, nonfiction, spoken	89,958	4741
Austronesian	Indonesian-PUD	News, wiki	19,446	1000
Austronesian	Indonesian-GSD	Blog, news	122,019	5598
Austronesian	Indonesian-CSUI	News, nonfiction	28,263	1030
Austroasiatic	Vietnamese-VTB	News	58,069	3323
Niger-Congo Atlantic	Wolof-WTB	Bible, wiki	44,258	2107
Afroasiatic	Arabic-PUD	News, wiki	20,747	1000
Afroasiatic	Arabic-NYUAD	News	738,889	19,738
Afroasiatic	Hebrew-IAHLT Twiki	Wiki	140,950	5039
Afroasiatic	Hebrew-HTB	News	160,195	6143
Afroasiatic	Coptic-Scriptorium	Bible, fiction, nonfiction	55,858	2163
Afroasiatic	Maltese_MUDT	News, nonfiction, legal, fiction, wiki	44,162	2074
Uralic	Estonian-EDT	Fiction, academic, news, nonfiction	438,245	30,968
Uralic	Finnish-TDT	Fiction, legal, news, blog, grammar-examples,	202,453	15,136
Uralic	Finnish-TDT	Poetry, medical, social, web	19,382	2122
Uralic	North Sami-Giella	News, nonfiction	26,845	3122
Uralic	Hungarian-Szeged	News	42,032	1800
Sino-Tibetan Sinitic	Chinese-GSDSimp	Wiki	123,291	4997
Iranian	Kurmanji_MG	Fiction, wiki	10,260	754
Iranian	Persian-PerDT	academic, blog, fiction, news, nonfiction, web	501,776	29.107
Iranian	Persian-Seraji	fiction, legal, medical, news, nonfiction, social, spoken	152,920	5997

## Appendix C. Morphological Richness (MAMSP) Values in Ascending Order

**Branch**	**Language**	**MAMSP**	**Branch**	**Language**	**MAMSP**
Vietic	Vietnamese	1	Romance	Romanian	1.1791
Sinitic	Chinese	1.0015	Slavic	Slovenian	1.1815
Japanese	Japanese	1.0488	Slavic	Croatian	1.1836
Germanic	Afrikaans	1.0687	Slavic	Belarusian	1.1928
Malayo-Polynesian	Indonesian	1.0829	Egyptian	Coptic	1.2001
Slavic	Russian	1.0877	Slavic	Serbian	1.202
Germanic	Norwegian-Nynorsk	1.0924	Baltic	Lithuanian	1.2162
Semitic	Hebrew	1.0982	Celtic	Scottish Gaelic	1.2195
Semitic	Arabic	1.1049	Finno-Ugric	North Sami	1.228
Finno-Ugric	Hungarian	1.1094	Greek	Greek	1.2391
Germanic	Swedish	1.1302	Basque	Basque	1.2416
Indo-Aryan	Hindi	1.131	Slavic	Czech	1.2435
Indo-Aryan	Urdu	1.1323	Celtic	Irish	1.2444
Germanic	Danish	1.1326	Dravidian	Telugu	1.2466
Slavic	Bulgarian	1.1344	Dravidian	Tamil	1.2474
Germanic	German	1.135	Finnic	Estonian	1.2503
Germanic	English	1.1375	Armenian	Armenian	1.2518
Iranian	Persian	1.1379	Atlantic	Wolof	1.2545
Germanic	Dutch	1.139	Romance	French	1.258
Romance	Spanish	1.1393	Mongolic	Buryat	1.27
Romance	Italian	1.1397	Finnic	Finnish	1.2985
Romance	Galician	1.1411	Iranian	Kurmanji	1.3164
Slavic	Slovak	1.1445	Turkic	Kazakh	1.3341
Romance	Portuguese	1.1458	Turkic	Turkish	1.36
Slavic	Polish	1.1496	Germanic	Gothic	1.4006
Romance	Catalan	1.1551	Indo-Aryan	Marathi	1.4344
Slavic	Ukrainian	1.1698	Turkic	Uyghur	1.4785
Slavic	Upper Sorbian	1.1729			

## Appendix D. Head-Final Dependency Counts and Percentages

**rank**	**deprel**	**head_final**	**head_final_pct_within_deprel**	**total_for_deprel**	**share_of_corpus_pct**
1	case	84,589	96.26	87,876	10.26
2	amod	49,069	82.06	59,797	6.98
3	punct	48,296	39.06	123,651	14.44
4	nsubj	45,450	78.33	58,025	6.78
5	det	44,673	95.65	46,705	5.45
6	advmod	31,399	75.89	41,373	4.83
7	cc	28,192	92.25	30,560	3.57
8	mark	20,803	97.08	21,429	2.50
9	obl	20,126	39.31	51,201	5.98
10	aux	14,811	75.79	19,542	2.28
11	cop	12,460	77.56	16,064	1.88
12	obj	11,085	29.28	37,864	4.42
13	nmod	9688	13.84	69,981	8.17
14	nummod	7669	72.76	10,540	1.23
15	advmod:emph	6119	83.67	7313	0.85
16	advcl	4488	39.71	11,301	1.32
17	expl:pv	3895	77.33	5037	0.59
18	compound	2965	65.82	4505	0.53
19	nsubj:pass	2948	77.62	3798	0.44
20	nmod:poss	2758	43.04	6408	0.75
21	obl:arg	2639	32.29	8174	0.95
22	aux:pass	2590	90.50	2862	0.33
23	nummod:gov	2455	98.79	2485	0.29
24	expl	1538	78.51	1959	0.23
25	xcomp	1267	12.18	10,404	1.21
26	amod:att	1205	99.18	1215	0.14
27	ccomp	1178	12.25	9615	1.12
28	discourse	1131	65.53	1726	0.20
29	expl:pass	974	81.30	1198	0.14
30	dep	972	24.87	3909	0.46
31	mark:prt	908	99.23	915	0.11
32	acl	894	14.68	6091	0.71
33	parataxis	815	15.36	5305	0.62
34	nsubj:cop	758	76.64	989	0.12
35	compound:lvc	744	98.41	756	0.09
36	iobj	698	34.22	2040	0.24
37	nmod:att	583	98.81	590	0.07
38	dislocated	448	80.43	557	0.07
39	advmod:mode	366	91.50	400	0.05
40	det:poss	362	99.18	365	0.04
41	case:gen	338	100.00	338	0.04
42	det:numgov	305	97.76	312	0.04
43	acl:relcl	300	3.71	8097	0.95
44	csubj	289	15.72	1838	0.21
45	vocative	239	64.25	372	0.04
46	obl:tmod	233	54.57	427	0.05
47	advmod:tlocy	210	92.11	228	0.03
48	clf:det	201	99.01	203	0.02
49	orphan	192	22.59	850	0.10
50	advmod:neg	180	96.26	187	0.02
51	compound:prt	161	20.46	787	0.09
52	nmod:tmod	152	92.12	165	0.02
53	aux:neg	138	92.62	149	0.02
54	aux:tense	131	99.24	132	0.02
55	case:acc	126	100.00	126	0.01
56	compound:nn	123	100.00	123	0.01
57	det:nummod	110	97.35	113	0.01
58	obl:mod	86	24.71	348	0.04
59	nmod:gobj	73	98.65	74	0.01
60	advmod:adj	65	42.48	153	0.02
61	nmod:unmarked	62	24.90	249	0.03
62	cc:preconj	58	98.31	59	0.01
63	obl:unmarked	57	33.33	171	0.02
64	nsubj:outer	52	96.30	54	0.01
65	det:predet	51	100.00	51	0.01
66	clf	43	15.25	282	0.03
67	obl:agent	41	8.47	484	0.06
68	expl:subj	40	86.96	46	0.01
69	mark:pcomp	39	100.00	39	0.00
70	expl:poss	34	89.47	38	0.00
71	nmod:desc	33	100.00	33	0.00
72	nmod:npmod	28	73.68	38	0.00
73	advmod:locy	28	90.32	31	0.00
74	nmod:obl	28	70.00	40	0.00
75	expl:impers	27	100.00	27	0.00
76	nmod:gsubj	26	100.00	26	0.00
77	reparandum	25	92.59	27	0.00
78	case:voc	24	100.00	24	0.00
79	obl:patient	22	100.00	22	0.00
80	list	20	4.44	450	0.05
81	obl:comp	18	11.04	163	0.02
82	xcomp:pred	15	1.78	842	0.10
83	compound:preverb	14	12.84	109	0.01
84	nsubj:nn	14	100.00	14	0.00
85	ccomp:obj	13	39.39	33	0.00
86	csubj:vsubj	13	100.00	13	0.00
87	case:adv	13	76.47	17	0.00
88	expl:comp	13	100.00	13	0.00
89	compound:affix	12	92.31	13	0.00
90	aux:caus	12	100.00	12	0.00
91	advmod:tmod	11	91.67	12	0.00
92	advmod:tto	10	100.00	10	0.00
93	csubj:pass	10	6.94	144	0.02
94	obl:appl	10	40.00	25	0.00
95	obj:lvc	9	31.03	29	0.00
96	obl:pmod	7	6.19	113	0.01
97	nmod:lmod	7	100.00	7	0.00
98	csubj:asubj	6	100.00	6	0.00
99	det:pmod	6	3.17	189	0.02
100	nsubj:caus	5	100.00	5	0.00
101	advmod:tfrom	5	83.33	6	0.00
102	obl:adj	5	29.41	17	0.00
103	nsubj:nc	5	100.00	5	0.00
104	csubj:cop	5	3.70	135	0.02
105	xcomp:ds	5	8.47	59	0.01
106	advmod:to	4	66.67	6	0.00
107	obj:appl	4	36.36	11	0.00
108	compound:svc	4	3.45	116	0.01
109	advcl:cond	4	100.00	4	0.00
110	cop:own	4	11.76	34	0.00
111	advcl:cmp	4	28.57	14	0.00
112	ccomp:obl	3	9.38	32	0.00
113	obl:lvc	3	50.00	6	0.00
114	parataxis:discourse	3	100.00	3	0.00
115	obj:caus	3	15.79	19	0.00
116	nsubj:xsubj	3	60.00	5	0.00
117	advcl:objective	3	4.69	64	0.01
118	csubj:outer	3	42.86	7	0.00
119	parataxis:insert	3	23.08	13	0.00
120	iobj:appl	2	66.67	3	0.00
121	obl:prep	2	0.93	215	0.03
122	acl:subj	2	1.41	142	0.02
123	obl:cmp	2	100.00	2	0.00
124	compound:redup	2	22.22	9	0.00
125	advcl:tcl	2	40.00	5	0.00
126	iobj:agent	2	66.67	3	0.00
127	obl:dat	1	0.88	114	0.01
128	advmod:que	1	25.00	4	0.00
129	advcl:pred	1	100.00	1	0.00
130	obl:with	1	2.04	49	0.01
131	obl:adv	1	100.00	1	0.00
132	compound:z	1	100.00	1	0.00
133	advmod:lmod	1	2.08	48	0.01
134	obj:agent	1	14.29	7	0.00

## Appendix E. Values of Word Order Flexibility (ENTR) in Ascending Order

**Branch**	**Languages**	**ENTR**	**Branch**	**Languages**	**ENTR**
Vietic	Vietnamese	0.247	Slavic	Polish	0.9109
Sinitic	Chinese	0.2985	Slavic	Ukrainian	0.9251
Indo-Aryan	Hindi	0.3311	Slavic	Upper Sorbian	0.9501
Japanese	Japanese	0.54	Egyptian	Coptic	0.9546
Iranian	Persian	0.5547	Mongolic	Buryat	0.9685
Finnic	Estonian	0.5821	Romance	Romanian	0.9823
Germanic	Norwegian-Nynorsk	0.6191	Slavic	Croatian	0.985
Celtic	Scottish Gaelic	0.6439	Romance	Catalan	0.9851
Indo-Aryan	Urdu	0.6515	Germanic	Gothic	0.9871
Germanic	English	0.6579	Finno-Ugric	Hungarian	1.0001
Turkic	Turkish	0.6888	Finno-Ugric	North Sami	1.0021
Germanic	Swedish	0.6923	Armenian	Armenian	1.0033
Semitic	Arabic	0.7068	Greek	Greek	1.0126
Romance	French	0.7073	Romance	Spanish	1.0194
Romance	Portuguese	0.7133	Romance	Italian	1.0198
Semitic	Hebrew	0.7134	Basque	Basque	1.0293
Romance	Galician	0.7136	Slavic	Bulgarian	1.1011
Turkic	Uyghur	0.7179	Germanic	German	1.144
Germanic	Danish	0.7565	Malayo-Polynesian	Indonesian	1.1602
Indo-Aryan	Marathi	0.7813	Germanic	Dutch	1.2634
Turkic	Kazakh	0.7869	Finnic	Finnish	1.3202
Dravidian	Telugu	0.7992	Slavic	Slovenian	1.3627
Dravidian	Tamil	0.8732	Slavic	Czech	1.3761
Germanic	Afrikaans	0.8782	Iranian	Kurmanji	1.3791
Slavic	Russian	0.8962	Baltic	Lithuanian	1.3881
Celtic	Irish	0.9009	Slavic	Slovak	1.4232
Slavic	Belarusian	0.9019	Atlantic	Wolof	1.4246
Slavic	Serbian	0.9059			

## Appendix F. Word Order Distribution of the 55 Languages

**Language**	**SVO %**	**SOV %**	**VSO %**	**VOS %**	**OVS %**	**OSV %**	**Language**	**SVO %**	**SOV %**	**VSO %**	**VOS %**	**OVS %**	**OSV %**
Armenian	0.6895	0.2120	0.0044	0.0049	0.0770	0.0122	Portuguese	0.8109	0.0298	0.0167	0.0401	0.1010	0.0015
Basque	0.5884	0.3039	0.0034	0.0170	0.0397	0.0476	Romanian	0.6111	0.2450	0.0033	0.0075	0.1305	0.0026
Bulgarian	0.8201	0.0544	0.0011	0.0200	0.0994	0.0050	Spanish	0.6210	0.1600	0.0110	0.0055	0.1960	0.0065
Belarusian	0.8544	0.0391	0.0000	0.0177	0.0621	0.0267	Catalan	0.6480	0.1548	0.0122	0.0049	0.1740	0.0061
Croatian	0.7344	0.0566	0.0326	0.0567	0.0898	0.0299	Greek	0.5846	0.0111	0.3011	0.0876	0.0113	0.0043
Czech	0.5080	0.0700	0.1010	0.1100	0.2000	0.0110	Maltese	0.2411	0.0000	0.6989	0.0411	0.0189	0.0000
Polish	0.7398	0.0440	0.0420	0.0520	0.1210	0.0012	Arabic	0.2310	0.0000	0.7284	0.0388	0.0018	0.0000
Russian	0.7446	0.0389	0.0110	0.0370	0.1387	0.0298	Hebrew	0.1810	0.0000	0.0650	0.0055	0.0110	0.0000
Slovak	0.4720	0.1260	0.0510	0.0780	0.2390	0.0340	Coptic	0.7258	0.2460	0.0040	0.0000	0.0000	0.0242
Slovenian	0.4730	0.1680	0.0390	0.0300	0.2490	0.0410	Marathi	0.1123	0.7944	0.0156	0.0211	0.0111	0.0455
Serbian	0.7527	0.0540	0.0133	0.0344	0.1122	0.0343	Hindi	0.0389	0.9231	0.0000	0.0016	0.0000	0.0364
Ukrainian	0.7345	0.0304	0.0156	0.0385	0.1433	0.0377	Persian	0.1488	0.8233	0.0012	0.0000	0.0017	0.0250
Upper Sorbian	0.7299	0.0493	0.0187	0.0322	0.1354	0.0345	Urdu	0.1520	0.7981	0.0110	0.0000	0.0025	0.0364
Danish	0.7968	0.0000	0.0026	0.0000	0.0891	0.1115	Indonesian	0.4818	0.0032	0.3451	0.0343	0.1254	0.0102
Dutch	0.5862	0.1000	0.0030	0.1000	0.0910	0.1198	Irish	0.2975	0.0007	0.6902	0.0106	0.0000	0.0009
English	0.7965	0.0000	0.0027	0.0000	0.0910	0.1098	Scottish Gaelic	0.2876	0.0000	0.7044	0.0080	0.0000	0.0000
Afrikaans	0.6714	0.0010	0.2007	0.0030	0.1230	0.0009	Japanese	0.0000	0.8277	0.0076	0.0060	0.0000	0.1587
German	0.4810	0.0016	0.3480	0.0300	0.1300	0.0094	Kurmanji	0.0910	0.6391	0.0033	0.0015	0.2630	0.0021
Gothic	0.6729	0.2087	0.0031	0.0374	0.0374	0.0405	Lithuanian	0.5349	0.1115	0.1925	0.1101	0.0446	0.0064
Norwegian-Nynorsk	0.6853	0.3133	0.0014	0.0000	0.0000	0.0000	Tamil	0.0000	0.6020	0.0000	0.0000	0.0772	0.3208
Swedish	0.7787	0.0000	0.0023	0.0000	0.1002	0.1188	Telugu	0.0220	0.7010	0.0000	0.0000	0.0360	0.2410
Estonian	0.8497	0.0088	0.0814	0.0091	0.0510	0.0000	Turkish	0.0343	0.7798	0.0042	0.0010	0.1510	0.0297
Finnish	0.4147	0.0906	0.1116	0.0000	0.1877	0.1954	Kazakh	0.0301	0.7661	0.0056	0.0020	0.1477	0.0485
Hungarian	0.5869	0.2243	0.0047	0.0000	0.1280	0.0561	Uyghur	0.0344	0.7886	0.0058	0.0021	0.1412	0.0279
North Sami	0.6075	0.2607	0.0579	0.0020	0.0599	0.0119	Buryat	0.2745	0.6009	0.0023	0.0017	0.1001	0.0205
French	0.7887	0.0820	0.0098	0.0000	0.1160	0.0035	Vietnamese	0.9511	0.0191	0.0000	0.0100	0.0000	0.0198
Galician	0.7581	0.2120	0.0075	0.0075	0.0050	0.0100	Chinese	0.9311	0.0344	0.0000	0.0000	0.0000	0.0345
Italian	0.6117	0.1662	0.0111	0.0059	0.2001	0.0050	Wolof	0.8734	0.0240	0.0115	0.0302	0.0255	0.0354
